# Modulating Mitochondrial DNA Heteroplasmy with Mitochondrially Targeted Endonucleases

**DOI:** 10.1007/s10439-022-03051-7

**Published:** 2022-08-24

**Authors:** Nikita Mikhailov, Riikka H. Hämäläinen

**Affiliations:** https://ror.org/00cyydd11 grid.9668.1 0000 0001 0726 2490 A.I.Virtanen Institute for Molecular Sciences, University of Eastern Finland, 70211 Kuopio, Finland

**Keywords:** mitoTALEN, Restriction endonuclease, Zinc finger nuclease, Oxidative phosphorylation, Genetic engineering, Gene therapy

## Abstract

Mitochondria, mainly known as energy factories of eukaryotic cells, also exert several additional signaling and metabolic functions and are today recognized as major cellular biosynthetic and signaling hubs. Mitochondria possess their own genome (mitochondrial DNA—mtDNA), that encodes proteins essential for oxidative phosphorylation, and mutations in it are an important contributor to human disease. The mtDNA mutations often exist in heteroplasmic conditions, with both healthy and mutant versions of the mtDNA residing in patients’ cells and the level of mutant mtDNA may vary between different tissues and organs and affect the clinical outcome of the disease. Thus, shifting the ratio between healthy and mutant mtDNA in patients’ cells provides an intriguing therapeutic option for mtDNA diseases. In this review we describe current strategies for modulating mitochondrial heteroplasmy levels with engineered endonucleases including mitochondrially targeted TALENs and Zinc finger nucleases (ZFNs) and discuss their therapeutic potential. These gene therapy tools could in the future provide therapeutic help both for patients with mitochondrial disease as well as in preventing the transfer of pathogenic mtDNA mutations from a mother to her offspring.

## Introduction

Mitochondria are double membrane bound organelles found in the cytoplasm of eukaryotic cells, that play a vital role in cellular energy metabolism. They break down nutrients and use oxygen to generate energy in the form of adenosine triphosphate (ATP) *via* oxidative phosphorylation (OXPHOS). In addition to their important role in energy production, mitochondria are involved in several additional cellular functions, including various signaling and biosynthetic pathways. The metabolic role of mitochondria reaches far beyond bioenergetics and today mitochondria are recognized as important metabolic hubs that catabolize energy from nutrients, produce precursors for macromolecules and manage metabolic waste.^63^ To date mitochondria have been shown to take part in biosynthesis of nucleotides, fatty acids, cholesterol, amino acids, glucose and heme.^63^ After the initial discovery that cytochrome C release from mitochondria can initiate a cascade that will lead to cell death,^38^ several additional signaling roles for mitochondria have been identified. Mitochondria generate, store and propagate many important signaling molecules including ROS, ATP and Calcium. Further, cellular signaling cascades can also be regulated by mitochondrial dynamics and morphology as well as by mitochondrial contacts with other organelles like endoplasmic reticulum (ER).^10^

## Mitochondrial Genome

Mitochondria contain their own bacterial like genome, which according to the endosymbiotic theory, stems from mitochondria originating from autotrophic bacteria engulfed by eukaryotic cells.^25^ However, after over two billion years of symbiosis, the modern mitochondria retain only a small part of the genes required to encode mitochondrial proteins.^25^ Human mtDNA is a circular double-stranded DNA molecule coding for 13 essential respiratory chain protein subunits, which are required for oxidative phosphorylation and ATP production. In addition to the protein coding genes, mtDNA also codes for the tRNAs and rRNAs needed for translation of these proteins.^14,69^ The rest of the nearly 1500 genes that are needed to build functional mitochondria are encoded by nuclear DNA^9,69^ and proteins transcribed from these genes in the cytoplasm are imported to mitochondria by translocases of the outer membrane and translocases of the inner membrane.^29^ For the cell to maintain its homeostasis, these two genomes must communicate and work tightly together in a synchronized manner.

Each mitochondrion contains multiple copies of mtDNA. On average, a somatic cell contains 1000 copies of mtDNA, with a range from 1 to 10 in a single mitochondrion.^60^ However, this varies drastically between different cell types, ranging from 1000 copies in liver and kidney cells to more than 100,000 copies in mature oocytes.^60^

**FIGURE 1:**
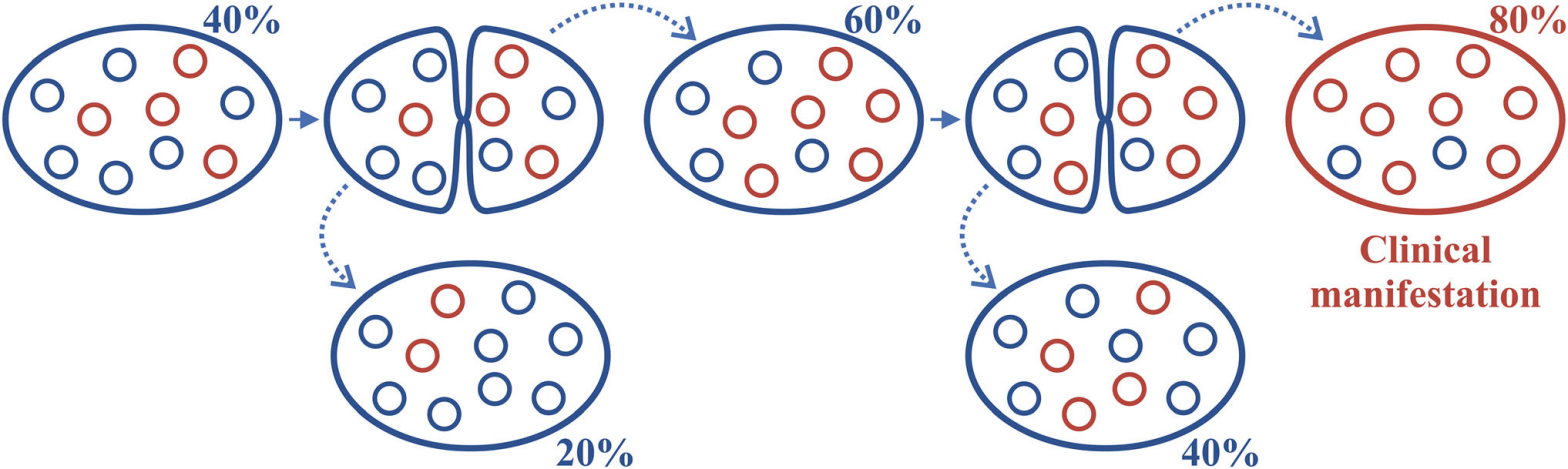
mtDNA heteroplasmy. A heteroplasmic cell contains two or more different mtDNA subtypes. Figure depicts heteroplasmic cells with wildtype (blue circles) and mutant (red circles) mtDNA. During cell division, mtDNA is randomly segregated between the daughter cells, which can lead to varying heteroplasmy levels in the progeny. For a mtDNA mutation to have functional consequences the mutation level should reach a threshold level, which may vary between different mutations. Figure schematically demonstrates heteroplasmy shift from 40% of mutant mtDNA in mother cell to 80% in daughter cell after two cell divisions.

A cellular state where all the mtDNA copies are identical is called homoplasmy, whereas the presence of two or multiple different mtDNA molecules within the same cell is termed heteroplasmy (Fig. 1). Heteroplasmy is observed in many patients harboring pathogenic mtDNA mutations and indeed majority of the human disease causing mtDNA mutations are heteroplasmic in nature.^37,68^ Whether the disease will manifest depends on the ratio of healthy and mutant mtDNA and the amount of mutant mtDNA should reach a critical threshold for a mitochondrial disease to have clinical manifestations.^13^ These thresholds vary between different mutations from 60% for large mtDNA deletions to 90% for some tRNA mutations.^35^ The thresholds for mutations to inflict functional effects can also vary between different cell types.^56^ The heteroplasmic nature of human disease causing mtDNA mutations leads to variable clinical phenotypes in the patients and poses challenges for the diagnosis of mitochondrial disease as for example the mutations levels of certain mtDNA mutations are known to decrease in blood, making diagnostics from blood samples difficult.^54^

## Mitochondrial Genetic Bottleneck

During the cell cycle, at cell division, mitochondria are normally considered to be distributed randomly between daughter cells, although selection for a specific subpopulation can occur in certain situations and cell types.^33^ For heteroplasmic mtDNA, this can lead to the progeny cells having significantly varying heteroplasmy levels.^35^ This feature of mtDNA inheritance is called mitotic segregation (Fig. 1).^13^ Rapid shifts in heteroplasmy levels between generations have been observed both in domestic animals and in human patients.^1,58^ This has led to the establishment of the mitochondrial genetic bottleneck hypothesis. This theory suggests that during transmission from one generation to another the mitochondria undergo a genetic bottleneck, involving either decrease in mtDNA copy number or selective, preferential amplification of a subset of a mtDNA type. The exact mechanism for this is still under debate. Also, the exact timing for this bottleneck has been under debate and existing data supports both germ cells and early preimplantation stage embryos.^8,11,17^ Further, this phenomenon may also take place later during development in certain cell types and could potentially partially explain the differences often seen in heteroplasmy levels between different tissues of a patient.^79^

## Mitochondrial DNA Diseases

The first studies of human disease-causing mtDNA mutations were reported in 1988^30,70^ and to date, more than 250 pathogenic mtDNA mutations have been identified^68^ The limited ability of mtDNA to self-repair, high mutation rate of the mitochondrial genome, generation of reactive oxygen species (ROS) during cellular respiration and close proximity of mtDNA to this as well as lack of protective histones makes mtDNA more susceptible to DNA damage than nuclear genome.^16^ Thus, sporadic mtDNA mutations are fairly common in population, and mitochondrial disease due to mutations in the mitochondrial DNA is considered a common cause of human inherited disease, with a prevalence of more than 1 in 5000 births.^61^ The maternal inheritance^23^ and polyploid nature of mtDNA, differentiates human mitochondrial genetics from Mendelian genetics and complicates the inheritance of mtDNA diseases. Homoplasmic mtDNA mutations are transmitted equally to all offspring, however, the transmission of heteroplasmic mtDNA mutations relies on various factors including chance and the bottleneck phenomenon in germ cells and during early development, leading to variable amounts of mutated mtDNA in different daughter cells.^66^ Thus, vast variation in mutation loads can be observed between family members and generations.^16,48^ MtDNA disorders are clinically heterogeneous with highly variable phenotypes and often manifest multisystemic effects. Usually, the most severely affected tissues are those that use most energy, namely the brain, the heart and skeletal muscle, but a mtDNA disease can manifest in any tissue in any severity and can have any age of onset. Further, the same mtDNA mutation can result in different outcomes in different families and even between family members.^24^ The most common brain disorders are encephalomyopathies like KSS (Kearns-Sayre syndrome), MERRF (myoclonus epilepsy and ragged- red fibers), MELAS (mitochondrial encephalomyopathy, lactic acidosis and stroke-like episodes), MILS (maternally inherited Leigh syndrome) and NARP (neuropathy, ataxia and retinitis pigmentosa).^13^ However, mtDNA mutations can also lead to muscle disease, cardiomyopathy, sensorineural hearing loss, retinopathy, optic atrophy, diabetes and gastrointestinal problems.^24^ The causative mutations can be single mtDNA re-arrangements, like large-scale deletions, point mutations in tRNA genes that affect translation of all mtDNA-encoded proteins, or point mutations in specific respiratory chain protein genes affecting only those specific proteins.^24^

The pathogenic mutations in mtDNA disrupt respiratory function and lead to respiratory chain deficiency.^9,13,14,69^ However, the drastically heterogeneous clinical phenotypes seen in patients with mtDNA mutations, varying even between patients carrying the same mutation, suggest that a sole respiratory chain and energy deficiency cannot fully explain the clinical outcome of the disease. Various organelle stress responses have thus been suggested to play a role in defining the functional outcome in patients’ cells, as these can vary between different tissues and patients depending on other innate metabolic factors.^64^ Some of the clinical variation may also be contributed to the varying heteroplasmy levels between patients and their tissues. The presence of a threshold, above which the mitochondrial disease starts to manifest, opens an idea that keeping the level of mutant mtDNA below the threshold could prevent disease onset, and in disease case, lowering the proportion of mutant mtDNA below this threshold could reverse the pathology. Thus, when seeking ways to treat mtDNA-related disorders, one interesting option is modulating mitochondrial heteroplasmy levels in patient cells.

## Replication and Repair of mtDNA

The minimal mtDNA replisome responsive for replication of the mitochondrial genome consists of the replicative DNA polymerase gamma (POLγ), the hexameric TWINKLE helicase that unwinds the double stranded mtDNA, the tetrameric mitochondrial single-stranded DNA-binding protein (mtSSB) and the mitochondrial RNA polymerase (POLRMT), which is required to produce RNA primers for POLγ.^72^ Mutations are much more frequent in mtDNA than in nuclear DNA.^74^ This was long considered to be the result of poor repair mechanisms for mtDNA, and DNA repair after replication and repair mechanisms beyond the exonuclease activity of POLγ were thought lacking. However, recent research has shown that mtDNA does have several effective repair mechanisms, with base excision repair being the most prominent one, yet the mechanisms for repairing double strand mtDNA breaks (mtDSBs) are indeed lacking.^74^ The presence of multiple copies of mtDNA within the mitochondria together with the circular nature of the mtDNA enables a way to resolve the double strand break problem by rapid degradation of linear mtDNA fragments. The resulting mtDNA depletion, i.e., reduction in copy number can be compensated by inducing replication of remaining native mtDNA.^59^

Indeed, induction of mtDSBs leads to rapid loss of linearized mtDNA.^45^ This was first shown in HEK293 cells expressing *Pst*I restriction enzyme. Expression of *Pst*I resulted in massive production of mtDSBs with subsequent and rapid loss in mtDNA copy number, 10% of mtDNA remained 8 h after *Pst*I digestion induction. The mtDSBs did not induce autophagy, mitophagy or apoptosis, suggesting that elimination of linear mtDNA fragments was not due to elimination of the mitochondria containing them. Knock-down of mitochondrial nucleases (EndoG, ExoG, MGME1, DNA2 and FEN1) by siRNA did not prevent degradation of linear mtDNA, leaving the mechanism for this degradation open.^45^

However, another study of mitochondrial nuclease activity in linear mtDNA degradation demonstrated that inactivation of MGME1 (mitochondrial genome maintenance exonuclease 1, but not ExoG, EndoG, DNA2, FEN1, APEX2, MRE11 or RBBP8) inhibited degradation of linear mtDNA, suggesting a role for MGME1 in this process.^49^ MGME1 is a metal-dependent single-stranded DNA binding exonuclease, with preference for 5’-3’ exonuclease activity, but capable also of digesting linear DNA.^65^ It has also been demonstrated that knockdown of POLγ or TWINKLE diminishes linear mtDNA degradation, suggesting that the mitochondrial replication machinery is also involved in this process.^49^ This rapid degradation of linear mtDNA fragments opens opportunities to specifically eliminate mtDNA subtypes simply by introducing cuts that will linearize the mtDNA fragments.

The idea of eliminating mutant mitochondrial genomes from heteroplasmic cells has raised vast attention. The current methods are based on mitochondrially targeted nucleases that will specifically bind and cut the mutant genome. The linearized DNA fragments are degraded, and compensatory replication of the remaining healthy genome results in regaining normal mtDNA copy number and reduction of the mutation level below the critical threshold. This shift in heteroplasmy level in favor of healthy mtDNA can restore functionality in the cells. Manipulation of mtDNA with transgenic technologies has been difficult and methods to introduce or correct specific mutations have been lacking until very recently (see chapter about mitochondria base editors). Thus, the nuclease-based systems have been the most prominent tool to manipulate mtDNA heteroplasmy. This review will discuss recent advances in this field.

## Shifting the Heteroplasmy Ratio in mtDNA Mutant Cells

Treatments for mitochondrial disease are still scarce and mostly symptomatic treatment is available for the patients. The high burden of mitochondrial diseases on the patients and on society and increasing attention to this problem have accelerated efforts to generate technologies for manipulation of the mtDNA. Transgenic technologies have been successfully used to manipulate nuclear DNA for decades. However, manipulation of the mtDNA has turned out to be much more difficult than manipulation of the nuclear genome, and viable methods to generate specific changes in mtDNA have been scarce. The multi-copy nature of the mitochondrial genome further complicates efforts to introduce specific changes in mtDNA. Thus alternative approaches for novel treatment options, like mitochondrial transplantation^40,76^ have been introduced to the field. Successful treatment of mitochondrial dysfunction with mitochondrial transplantation is however not yet proven, requires several repetitive interventions, and opens a discussion on safety and compatibility of the recipient cell and donor mitochondria,^27^ leaving, at least at the current stage of technology,^75^ room for the actual DNA editing technologies in the therapy development. In addition, mitochondrial transplantation does not remove the source of the problem, mutant mtDNA, but rather just temporarily increases the amount of healthy mitochondria. Whereas gene-editing approaches treat the problem at the source, tackling the disease-causing mutations in mtDNA and could therefore be more profitable over the long run.

**FIGURE 2:**
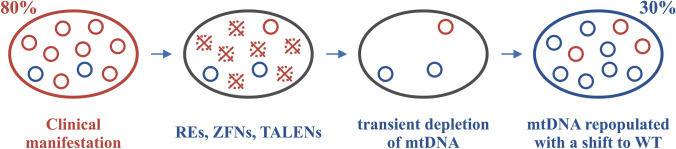
Nuclease based manipulation of heteroplasmy levels. Mutation specific nucleases are targeted to mitochondria, where they cut and linearize mutant mtDNA molecules. The linear fragments are degraded by the cells’ innate machinery, leading to a shift in heteroplasmy level and an increase in the relative proportion of healthy mtDNA. After compensatory replication, the amount of mtDNA is restored.

Nucleases, both endo- and exonucleases, can have their place in modifying mtDNA. While exonucleases, like MGME1, can take part in DNA repair breaking the free ends of a polynucleotide chain, and participate in degradation of linear DNA fragments, endonucleases can initiate the linearization process by cleaving circular mitochondrial genomes. The nuclease-based approaches all build on the rapid degradation of linear mtDNA fragments and share a common idea of specifically cutting and linearizing the mutant mtDNA with endonucleases targeting only the mutant allele.^47^ This leads to a transient reduction in the total number of mtDNA but with a shift in the proportion of the mutant to healthy mtDNA variant (Fig. 2).

**FIGURE 3:**
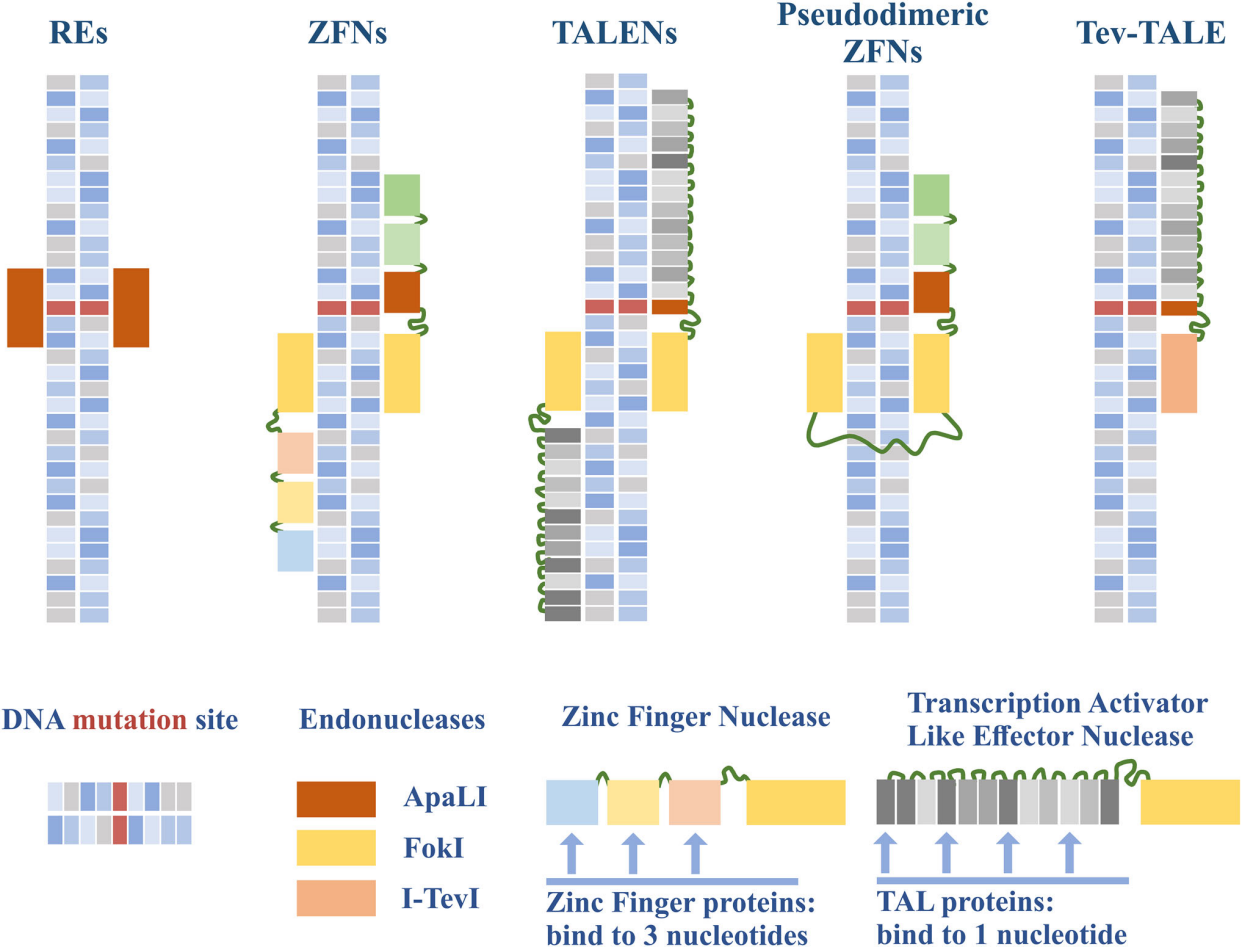
Different nuclease types used in manipulation of mitochondrial heteroplasmy and their binding manner. Figure depicts the most common constructs—restriction endonucleases (REs), zinc finger nucleases (ZFNs) and transcription activator like effector nucleases (TALENs). In addition, alternatives like pseudodimeric ZFNs and monomeric nucleases (Tev-TALE) are presented.

To date there are several nuclease-based technologies available to mitigate mitochondrial heteroplasmy: restriction endonucleases (REs), zinc finger nucleases (ZFNs) and transcription activator like effector nucleases (TALENs) are among them^76^ (Fig. 3). High hopes are set for the possibility of elimination of mutant mtDNA in heteroplasmic cells using these different nucleases and for the use of these techniques in therapeutic interventions.^5,19^ However, while the levels of heteroplasmic mutations can be manipulated through these nuclease-based methods, the method is inefficient against homoplasmic mutations and thus somewhat restricted.

## Manipulating Heteroplasmy Levels with Restriction Endonucleases

The first attempts to manipulate mutation levels in heteroplasmic cells with endonucleases utilized natural bacteria derived restriction enzymes. In a study by Reddy et al. a heteroplasmic NZB/BALB mouse model, carrying two different normal mtDNA haplotypes (NZB and BALB), was used to prove the ability of *ApaL*I restriction enzyme to specifically reduce one of the mtDNA haplotypes (BALB haplotype contains a recognition site for *ApaL*I, while NZB does not) and to prevent transmission of a specific mtDNA haplotype to the offspring.^55^ To achieve this the authors combined *ApaL*I endonuclease with ATP5B mitochondrial targeting sequence and with ATP5B 5’ and 3’ UTRs to promote mitochondrial import from mitochondria-associated ribosomes. This construct was injected to mouse oocytes, where it resulted in a significant reduction of the BALB genotype in 48 h and led to a subsequent increase in the NZB genotype.

Although restriction enzymes have a handful of advantages, including remarkably high recognition accuracy and cutting efficacy,^76^ there are only a limited number of enzymes naturally available with limited recognition sites. Additionally, the binding site for most restriction enzymes is quite short and usually varies from 4 to 6 base pairs, although it may be up to 12 base pairs in some cases,^31^ and the short DNA recognition sequence reduces specificity of binding. Further, only a few pathogenic mtDNA mutations have respective recognition sites for naturally occurring restriction enzymes. *Xma*I can cleave the m.8993T > G mutation that underlies NARP and MILS,^55^ however, the majority of the human disease causing mtDNA mutations lack specific restriction enzyme target sequences. As restriction enzymes cannot be artificially manipulated,^52,76^ they are quite limited tool for heteroplasmy modulation.

Fortunately, other modifiable nuclease types, that can be engineered to cleave a specific mtDNA subtype, exist. These include artificial zinc finger nucleases (ZFNs) and transcription activator like effector nucleases (TALENs). Both of these can be engineered to target mitochondria and specifically recognize specific mutations. These approaches are considered in following sections.

## Manipulating Heteroplasmy with Zinc Finger Nucleases

The ZFN approach shares some aspects of the restriction enzyme mechanism.^76^ It consists of a modifiable DNA recognition domain, a zinc finger protein, bound to a non-specific *Fok*I restriction endonuclease. The *Fok*I endonuclease performs DNA cleavage only as a dimer,^62^ requiring binding of one zinc finger to 5′–3′ and another to 3′–5′ DNA sequence around the desired cutting site. For efficient cutting, the spacer area between the binding sites for the two DNA binding domains of a ZFN, can be between 4 to 6 base pairs and increasing the length of the spacer dramatically reduces efficacy of targeting.^39^

The DNA recognition domain of a ZFN consists of three zinc finger proteins (ZFP), each binding to a sequence of 3 nucleotides, resulting in a 9-nucleotide recognition sequence per ZFN and in total of 18 specific nucleotides to be recognized by the ZFN dimer to perform double strand DNA break.^39^ This supplies high recognition specificity for the system.

A single ZFP has ββα secondary structure, and specificity of the DNA binding is provided by hydrogen bonds of only four contact amino acids found at positions 1, 2, 3, and 6.^34^ Different combinations of these key amino acids can be designed to bind nearly every combination of a sequence of 3 nucleotides. This was proven both experimentally and by *in silico* modelling in a recent comprehensive study.^51^

Systemic online screening resources for identification of ZFP binding sites^7^ allow prediction of the nucleotide binding sequence of a given ZFP, or *vice versa* to find a specific amino acid sequence that will bind to a given sequence of three base pairs.

To provide effective delivery of the ZFPs to mitochondria a mitochondrial targeting sequence (MTS) should be attached to the construct. Further, to avoid toxic effects by unintentional localization of the ZFPs in the nucleus, and to facilitate the re-export from nucleus in case they appear there, ZFPs can also be merged with nuclear export signals (NES). This will result in MTS-ZFPs-NES construct with highly specific distribution only in mitochondria, and eliminate off-target effects to nuclear genome.^41^

**FIGURE 4:**
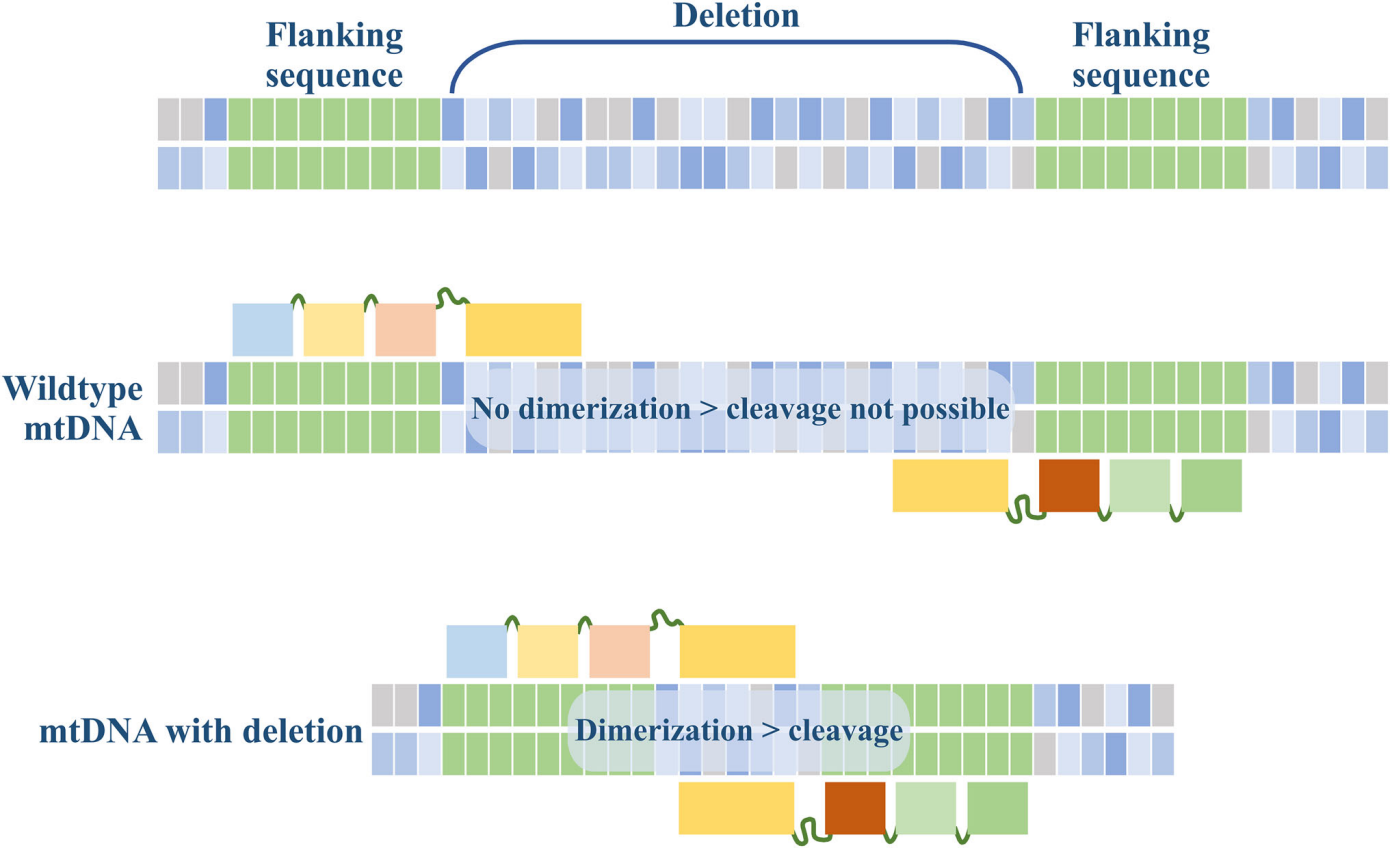
Targeting strategy for large scale mtDNA deletions. The top panel presents full-length mtDNA of the deletion region. Dimeric ZFNs are designed to bind to sequences of mtDNA flanking the deletion. In the case of wildtype mtDNA, the long distance between the bound ZFNs prevents dimerization of nucleases (middle panel). In the case of mutant mtDNA with deletion, the ZFNs close proximity to each other allow dimerization and mtDNA cleavage takes place (bottom panel).

Mitochondrially targeted ZFNs have been used to modify heteroplasmy in wild type human osteosarcoma cells and in cybrid cells bearing either the m.8993T > G point mutation associated with NARP and MILS or a common large mtDNA deletion, a 5-kilobase deletion present in 30% of patients with mtDNA deletions associated with chronic progressive external ophthalmoplegia (cPEO), Kearns-Sayre syndrome (KSS) and Pearson syndrome.^21^ In the case of the point mutation, one ZFN monomer attaches to the sequence containing the point of mutation, excluding binding to the healthy allele, and another ZFN binds to an adjacent region on the corresponding strand allowing dimerization of the *Fok*I endonucleases (Fig. 3). Specificity for targeting the large deletion in mtDNA is different (Fig. 4). In this case, both ZFN monomers bind unspecifically to both wildtype and mutant mtDNA to regions flanking the deletion, but *Fok*I dimerization takes place only in the mtDNA molecules with the deletion. Whereas in the case of the full-length mtDNA, the *Fok*I monomers are separated by the 5 kb deletion region and thus are not able to dimerize and cut DNA.

A single round of nuclease treatment may not lead to a substantial enough decrease in the mutation level to enhance functionality. However, to afflict prominent shifts in heteroplasmy, several repetitive ZFNs transfections can be performed. Gammage and others treated heteroplasmic m.8993T > G mutant cells four consecutive times with mutation targeting ZFNs, with 28 days between iterations to allow restoration of the mtDNA copy number between treatments. This led to reduction of mutant mtDNA from the 80% in the starting human osteosarcoma 143B cybrids to 7% in the final treated cells.^19^

An alternative approach for designing ZFNs was shown by Minczuk and colleagues.^42^ Engineering a pair of ZFPs targeting two corresponding mtDNA strands around a spacer sequence is not always possible, for example due to the lack of specificity. In these situations, it is possible to construct a single-chain quasi-dimeric ZFN, where one ZFN contains both *Fok*I endonuclease units required for mtDNA cleavage. This approach was used to create MTS-ZFPs-NES-*Fok*I-*Fok*I construct targeting the m.8993T > G mutation and was successfully tested *in vitro* in NARP cell cybrids.

## Mitochondrially Targeted TALENS

The general principle of mitoTALEN action is similar to the one of the mitoZFNs. Two TALEN constructs, each consisting of a DNA recognition domain and a non-specific *Fok*I endonuclease, bind to both 5′–3′ and 3′–5′ DNA strands, so that *Fok*I can dimerize and cut the DNA.^76^ The length of the spacer domain between the TALENs where the *Fok*I dimer cleaves the DNA double strand is however significantly longer and may vary between 12 to 20 base pairs.^18^ Despite the similarity of TALENs and ZFNs action, the TAL protein domain responsible for DNA sequence recognition is composed differently. The DNA recognition domain of a single TALEN consists of around 20 TAL proteins (modules) each responsible for binding only to one nucleotide,^6,46^ which minimizes off-target cleavage and makes the TALENs highly specific^28^ (Table 1 summarizes main features of different mitochondrially targeted nucleases). The amino acid sequence of a single TAL protein and its secondary structure are highly conservative. A TAL protein consists of 33–35 amino acids and folds into two α-helices—short and long ones.^12^ Whereas most of the amino acids of the TAL protein are conserved, two amino acids, located at positions 12 and 13 and referred to as repeat variable diresidues (RVDs), can vary and define the specificity of nucleotide recognition.^6,46^

**TABLE 1:** Summary of the main features of mitochondrially targeted nucleases.

	mitoRE	mitoZFN	mitoTALEN
Modifiable	No^52,76^	Yes^51^	Yes^15^
Average binding site of a single nuclease	4–6^31^	9^39^	≈ 20^6,46^
Binding site of a single recognition unit	n/a	1ZFP—3 bp^39^	1TALE—1 bp^6,46^
Advantages	Small	Small, high specificity	Easy to design, high specificity^28^
Disadvantages	Limited specificity, limited area of application due to unmodifiable nature^52,76^	Difficult to design	Large

This modular nature of TALENs and the fact that each module corresponds only to one nucleotide, whereas one ZFP module should bind three nucleotides simultaneously, makes TALEN a perfect tool for gene therapy.^18^ The only limitation for the target DNA sequence is that it should start with a thymine nucleotide.^18,46^ As for the ZFNs, tools for prediction of binding sites for specific RVDs, designing TALENs for a given DNA sequence and more are available online.^15,67^ The strategy for selecting binding regions and engineering mitochondrially targeted TALENs (mitoTALENs) for heteroplasmy modulation is similar to the one of ZFNs including merging the TALENs to mitochondrial targeting and nuclear export signals. The efficiency of the mitoTALENs to shift heteroplasmy levels was demonstrated in human osteosarcoma cells.^5^ The mitoTALENs were designed to bind mtDNA sequences flanking the site of the common 5 kb deletion region. As described for the ZFNs, in the mtDNA molecules with the deletion the binding of the TALENs to the mtDNA enable dimerization of *Fok*I and subsequent mtDNA cleavage, whereas binding to the full-length mtDNA molecules does not allow dimerization of the *Fok*I enzymes. Two days after transfection a fraction of cells expressing both right and left TALENs demonstrated a two-fold increase in the percentage of the wild type mtDNA, caused by reduction of the mutant mtDNA content. Two weeks after transfection, a small decrease in total mtDNA content was still detected, however, it was accompanied by mtDNA content repopulation by the wild-type haplotype. When transfected to homoplasmic osteosarcoma cells bearing only full-length mtDNA, the mitoTALENs did not alter mtDNA copy number, which demonstrated the specificity of these mitoTALENs for the deletion molecule. In another study mitoTALENS were shown to efficiently target a heteroplasmic ND6 point mutation, m.14459G > A responsible for Leber’s hereditary optic neuropathy.^5^ One TALEN was designed to bind a wild-type sequence adjacent to the mutation, whereas another one was targeted to the mutation site, allowing binding and dimerization of the *Fok*I endonuclease only in the presence of the mutation. Two days after transfection, the proportion of wild-type and mutant mtDNA was reversed with the wild-type haplotype now dominating.

A third study^28^ demonstrated the design and efficiency of mitoTALENs targeting point mutations associated with MERRF (m.8344A > G in the tRNA^Lys^ gene) or MELAS and Leigh syndrome (m.13513G > A in the ND5 gene) in cybrids of mtDNA-free Rho0 osteosarcoma cells fused with dermal fibroblasts carrying these point mutations. This study showed that successful transfection of mitoTALENs led to nearly total elimination of mutant mtDNA from the cells, and the level of the wild-type haplotype remained close to 100% for the whole 30 days follow-up period.

The potential of mitoTALENs for targeting one of the most common human disease-causing mtDNA mutations, was demonstrated in iPSCs (induced pluripotent stem cells) derived from patients with the m.3243A > G point mutation [targeting tRNA^Leu(UUR)^]. This mutation can lead to variable phenotypes resulting in either MIDD syndrome (maternally inherited diabetes and deafness), MELAS syndrome or cardiomyopathy in different patients. After successful reduction of the mutation level by the mitoTALENs, neural progenitor cells derived from the TALEN treated iPSCs demonstrated enhanced and normal respiratory function, proving that the TALEN treatment was able to reduce mutation levels and recover functionality of the cells.^77^

## Monomeric Nucleases

The requirement of endonuclease dimerization for DNA cleavage activity has its advantages, e.g., allowing targeting of large-scale deletions as described above, and higher specificity of the action. However, the need for dimerization doubles the load of the cargo that should be delivered to mitochondria to induce mtDSBs. To enhance the transfection efficiency, especially for putative *in vivo* applications, the use of monomeric nucleases for manipulation of mtDNA heteroplasmy has been studied.

The following construct was used as a substitution for dimeric TALENs: mitochondria localization sequence (MLS), TAL DNA binding domain and monomeric endonuclease I-*Tev*I. The resulting mitoTev-TALE nuclease reduced mutant mtDNA load in patient-derived cybrid cells carrying the m.8344A > G point mutation, responsible for MERRF syndrome.^50^ Transfected cells showed increase in relative wildtype mtDNA haplotype already the next day after transfection. Further, the treatment restored mitochondrial function in the transfected cells, and mitochondrial respiration was enhanced overall, including maximal respiratory capacity and ATP-linked respiration. This study showed that also monomeric nucleases are effective in shifting mtDNA heteroplasmy levels and result in functional outcome. Thus, with less cargo and easier delivery to the recipient cells, they may have advantages over the dimeric nucleases in some applications.

## *In Vivo* Applications

Shifting mtDNA heteroplasmy levels *in vivo* presents an added difficulty in terms of delivery of the enzymes to the site of their action. Viral delivery of the nucleases has proven efficient and shown beneficial effects on heteroplasmic animals. Adeno-associated virus type-6 (AAV6) and hepatotropic adenovirus type-5 (Ad5) were used for intravenous delivery of mitochondrially targeted *ApaL*I restriction enzyme respectively to the heart and liver of NZB/BALB mouse model and resulted in reduced level of the *ApaL*I containing BALB haplotype.^4^ In another study, a single intravenous or intraperitoneal injection of adeno-associated virus type-9 (AAV9) carrying mitochondrially targeted *ApaL*I enzyme to the NZB/BALB newborn mice led to a prominent and long lasting (up to 24 weeks after injection) increase in NZB haplotype in skeletal muscles and heart of these mice.^3^

Further, restriction enzyme *ApaL*I delivered to the one-cell embryos of NZB/BALB mice^55^ led to significant decrease in the BALB haplotype (containing *ApaL*I site) after cultivation of embryos to the blastocyst stage. Thereafter, these blastocysts were transferred to a pseudo pregnant mouse that gave birth to live pups (F1 generation) with reduced number of BALB mtDNA copies in biopsies of brain, muscle, heart, and liver, demonstrating systemic clearance of the selected mtDNA haplotype. Furthermore, next F2 generation retained this heteroplasmy shift towards NZB mtDNA haplotype. This study proved efficacy of the restriction endonucleases in pre-implantation stage gene editing with positive and sustainable shift in heteroplasmy that was carried over to the next generations.

Besides restriction enzymes, also the modifiable nucleases have been shown capable of shifting mtDNA heteroplasmy levels *in vivo*. Mitochondrially targeted ZFNs were delivered by AAV9 to mice with mtDNA point mutation m.5024C > T in the tRNA^Ala^ gene in cardiac tissue, where they led to a shift in heteroplasmy that progressed over time with the highest shift registered 65 days after exposure to the nucleases.^22^

Engineered mitoTALENs were used to modify single-point mutation heteroplasmy in mice carrying the same m.5024C > T mutation in the tRNA^Ala^ gene.^2^ AAV9-mitoTALENs were delivered either intramuscularly, intravenously, or intraperitoneally and all three delivery approaches resulted in reduction of the mutant mtDNA both in heart and skeletal muscle.

Despite the successful *in vivo* editing studies, the construct size and targeting efficiency still pose challenges for *in vivo* applications. To overcome several of the remaining hurdles in *in vivo* editing, Zekonyte et al. adapted the *I-Cre*I homing meganuclease based gene editing platform, known as ARCUS, to mitochondrial genome.^78^ The *I-Cre*I is a homodimeric endonuclease that associates with a palindromic 22-bp double stranded sequence and produces a double strand break (DSB). The resulting mitoARCUS meganuclease has a relatively small size and can work as a monomere, which makes it more efficient in *in vivo* applications. Further, it can recognize sequences differing only at a single base, making it highly flexible and selective.^78^ The authors further proved the effectiveness of this system on eliminating mutant mtDNA by delivering MitoARCUS targeting a m.5024C > T mutation in the tRNA^Ala^ gene intravenously using AAV9 in mice carrying this mutation. The amount of mutant mtDNA was rapidly reduced and subsequent restoration of the total mtDNA levels were also shown to quickly take place.^78^

## Preventing Transmission of mtDNA Disease

Besides recent efforts on trying to develop treatment strategies for improving bioenergetics and cellular function in the mtDNA patients, another topic, that has been under enormous research in recent years, revolves around transmission of mtDNA mutations to the next generation and ways to prevent transmission of disease from the mother to her offspring. *In vitro* fertilization (IVF) together with preimplantation genetic diagnosis (PDG) have been utilized to allow implantation of the embryos with the lowest mtDNA mutation loads.^57^ However, this technology is not always successful as in many cases all embryos still carry high mutation amounts and further, girls born through this technology are still at risk of transmitting the disease to their offspring.^53^

Mitochondrial nucleases could provide a way to enhance the PGD applications and allow reduction or even complete elimination of the mtDNA mutations from heteroplasmic embryos before implantation. However, this technique will only benefit families with heteroplasmic mutations.

## Therapeutic Potential of mtDNA Heteroplasmy Modulation

Despite decades of clinical and genetic studies, there are still no curative therapeutics for patients with mtDNA disease, thus, the clinical potential of gene therapy is considerable. Both mitoTALENs and mitoZFNs have been proven successful in altering heteroplasmy levels *in vitro* and *in vivo*. However, before the technology can be moved to the clinic, several aspects still need attention.

While viral delivery has been successfully used to deliver nucleases to animal cells *in vivo*,^2–4,22,55^ the current recombinant AAVs and their manufacturing methods are still lacking and when scaled to human level, the dosing used in these preclinical animal studies would result in potentially unsafe viral levels.^71^ Thus, more advanced delivery methods, or more efficient nucleases that would allow downscaling the nuclease encoding AAV dose, are needed. Another putative safety issue is immunogenicity. The immunogenicity of viral delivery vehicles is an issue in all gene therapy approaches and has been studied actively.^43^ Further, for mitochondrially targeted nucleases also the nucleases themselves could potentially pose immunogenic issues. The *Fok*I catalytic domain is from bacterial origin and could activate immunogenic response and in the case of mitoTALENs, the same can be true for the TAL domain which also is of bacterial origin.^32^ These questions on putative immune responses against the nucleases need to be addressed before the technology can reach the clinic. The third important question in all gene therapy approaches is putative off-target effects. For mitochondrial nuclease-based therapy, this however does not seem to be a significant issue, as the nucleases can be efficiently targeted to mitochondria, using a combination of mitochondrial targeting and nuclear export signals (NES), hindering any possible off-target effects on the nuclear genome.^41^ Further, while off-target effects are likely to take place in mitochondria, the targeted linearized mtDNA molecules are rapidly degraded, and thus will not result in permanent modification of the mtDNA but just temporary mtDNA depletion, which will recover quickly. However, before this approach can be taken to the clinic, all these safety aspects warrant additional studies and more detailed knowledge on what actually takes place in the cells.

## Mitochondrial Base Editors

Manipulation of mitochondrial DNA with traditional transgenic technologies or the CRISPR based approaches has been extremely difficult, leaving the nuclease-based systems as the main method to modulate mtDNA heteroplasmy. The major hurdle in the CRISPR methodology has been the difficulty to introduce DNA or RNA into mitochondria.^20^ A breakthrough in this field, came when Mok et al. described a purely protein-based base-editor, which they named DddA.^44^ They used an interbacterial toxin that catalyzes deamination of cytidines within double-stranded DNA (dsDNA) and engineered a non-toxic split-protein, which is inactive until the two halves are brought together. The targeting to the desired sequence was achieved through binding the split protein halves to programmable DNA binding motifs.^44^ Fusing the split-DddA halves to transcription activator-like effector array proteins for targeting and a uracil glycosylase inhibitor further resulted in DddA-derived cytosine base editors (DdCBEs) that catalyse C·G-to-T·A conversions. These are RNA-free, which allows delivery of all components to mitochondria and targeting mtDNA with high specificity.^44^ The authors demonstrated efficiency of this system by targeting pathogenic mtDNA mutations in human cells, which resulted in correction of the mutations and restoration of function in the cells.^44^

The efficiency of the DdCBE system in manipulating mitochondrial genomes has further been proven in studies where mice with specific C-to-T mtDNA mutations were generated and the mutations were transmitted to the next generations. These studies prove that the DdCBE platform now allows generation of transgenic animal models for mtDNA disease which have previously been lacking.^26,36^

The methodology is ground breaking and has raised high hopes, however, a drawback to the use of the system may arise from unspecificity and off-target effects, as it was soon reported that the mt-DdCBEs lead to off-target effects both in the mitochondrial and nuclear genomes. Low frequency off-target changes in mtDNA seem to be fairly common but even more worrying is the substantial amount of off-target single-nucleotide changes seen in the nuclear genome,^73^ which may limit the usability of the mt-DdCBEs at least for therapeutic purposes.

### Conclusions

Mitochondrial disease due to mtDNA mutations are a heterogeneous group of diseases that can manifest at any age, affect almost any tissue or be multisystemic, and have drastically varying clinical outcomes. The inheritance and clinical outcome are significantly complicated by the multicopy nature of the mitochondrial genome and heteroplasmic state of most human disease-causing mutations. Vast research around mtDNA maintenance, inheritance and segregation of mitochondria during cell division, in different cell types and during development have advanced our understanding of mtDNA disease, yet treatment options for the patients are still scarce. Recent advances in gene technology have raised hopes for gene therapy options in mitochondrial disease, with mitochondrially –targeted nucleases as one of the most prominent options. However, before moving the approach to the clinic, several safety issues regarding *in vivo* delivery and immunogenicity still need addressing.

## References

[1] Ashley, M. V., P. J. Laipis, and W. W. Hauswirth. Rapid segregation of heteroplasmic bovine mitochondria. *Nucleic Acids Res.* 17:7325, 1989. 2798094 10.1093/nar/17.18.7325 PMC334812

[2] Bacman, S. R., J. H. K. Kauppila, C. V. Pereira, N. Nissanka, M. Miranda, M. Pinto, S. L. Williams, N. G. Larsson, J. B. Stewart, and C. T. Moraes. MitoTALEN reduces mutant mtDNA load and restores tRNAAla levels in a mouse model of heteroplasmic mtDNA mutation. *Nat. Med.* 24(11):1696–1700, 2018. 30250143 10.1038/s41591-018-0166-8 PMC6942693

[3] Bacman, S. R., S. L. Williams, D. Duan, and C. T. Moraes. Manipulation of mtDNA heteroplasmy in all striated muscles of newborn mice by AAV9-mediated delivery of a mitochondria-targeted restriction endonuclease. *Gene Ther.* 19:1101–1106, 2012. 22130448 10.1038/gt.2011.196 PMC3410960

[4] Bacman, S. R., S. L. Williams, S. Garcia, and C. T. Moraes. Organ-specific shifts in mtDNA heteroplasmy following systemic delivery of a mitochondria-targeted restriction endonuclease. *Gene Ther.* 17:713–720, 2010. 20220783 10.1038/gt.2010.25 PMC3175591

[5] Bacman, S. R., S. L. Williams, M. Pinto, S. Peralta, and C. T. Moraes. Specific elimination of mutant mitochondrial genomes in patient-derived cells by mitoTALENs. *Nat. Med.* 19(9):1111–1113, 2013. 23913125 10.1038/nm.3261 PMC4153471

[6] Boch, J., H. Scholze, S. Schornack, A. Landgraf, S. Hahn, S. Kay, T. Lahaye, A. Nickstadt, and U. Bonas. Breaking the code of DNA binding specificity of TAL-type III effectors. *Science*. 326:1509–1512, 2009. 19933107 10.1126/science.1178811

[7] B1H screens of C2H2-ZF domainsat. http://zf.princeton.edu/b1h/index.html

[8] Cao, L., H. Shitara, T. Horii, Y. Nagao, H. Imai, K. Abe, T. Hara, J. I. Hayashi, and H. Yonekawa. The mitochondrial bottleneck occurs without reduction of mtDNA content in female mouse germ cells. *Nat. Genet.* 39:386–390, 2007. 17293866 10.1038/ng1970

[9] Chan, D. C. Mitochondria: dynamic organelles in disease, aging, and development. *Cell*. 2006. 10.1016/j.cell.2006.06.010. 16814712 10.1016/j.cell.2006.06.010

[10] Chandel, N. S. Mitochondria as signaling organelles. *BMC Biol.* 12:1–7, 2014. 24884669 10.1186/1741-7007-12-34 PMC4035690

[11] Cree, L. M., D. C. Samuels, S. C. De Sousa Lopes, H. K. Rajasimha, P. Wonnapinij, J. R. Mann, H. H. M. Dahl, and P. F. Chinnery. A reduction of mitochondrial DNA molecules during embryogenesis explains the rapid segregation of genotypes. *Nat. Genet.* 40:249–254, 2008. 18223651 10.1038/ng.2007.63

[12] Deng, D., C. Yan, X. Pan, M. Mahfouz, J. Wang, J. K. Zhu, Y. Shi, and N. Yan. Structural basis for sequence-specific recognition of DNA by TAL effectors. *Science*. 335:720–723, 2012. 22223738 10.1126/science.1215670 PMC3586824

[13] DiMauro, S., and G. Davidzon. Mitochondrial DNA and disease. *Ann. Med.* 2005. 10.1080/07853890510007368. 16019721 10.1080/07853890510007368

[14] DiMauro, S., and E. A. Schon. Mitochondrial respiratory-chain diseases. *N. Engl. J. Med.* 348:2656–2668, 2003. 12826641 10.1056/NEJMra022567

[15] Doyle, E. L., N. J. Booher, D. S. Standage, D. F. Voytas, V. P. Brendel, J. K. Vandyk, and A. J. Bogdanove. TAL Effector-Nucleotide Targeter (TALE-NT) 2.0: tools for TAL effector design and target prediction. *Nucleic Acids Res.* 40:W117–W122, 2012. 22693217 10.1093/nar/gks608 PMC3394250

[16] Druzhyna, N. M., G. L. Wilson, and S. P. LeDoux. Mitochondrial DNA repair in aging and disease. *Mech. Ageing Dev.* 129:383–390, 2008. 18417187 10.1016/j.mad.2008.03.002 PMC2666190

[17] Freyer, C., L. M. Cree, A. Mourier, J. B. Stewart, C. Koolmeister, D. Milenkovic, T. Wai, V. I. Floros, E. Hagström, E. E. Chatzidaki, R. J. Wiesner, D. C. Samuels, N. G. Larsson, and P. F. Chinnery. Variation in germ line mtDNA heteroplasmy is determined prenatally but modified during subsequent transmission. *Nat. Genet.* 44:1282, 2012. 23042113 10.1038/ng.2427 PMC3492742

[18] Gaj, T., C. A. Gersbach, and C. F. Barbas. ZFN, TALEN, and CRISPR/Cas-based methods for genome engineering. *Trends Biotechnol.* 31:397–405, 2013. 23664777 10.1016/j.tibtech.2013.04.004 PMC3694601

[19] Gammage, P. A., E. Gaude, L. Van Haute, P. Rebelo-Guiomar, C. B. Jackson, J. Rorbach, M. L. Pekalski, A. J. Robinson, M. Charpentier, J. P. Concordet, C. Frezza, and M. Minczuk. Near-complete elimination of mutant mtDNA by iterative or dynamic dose-controlled treatment with mtZFNs. *Nucleic Acids Res.* 44:7804–7816, 2016. 27466392 10.1093/nar/gkw676 PMC5027515

[20] Gammage, P. A., C. T. Moraes, and M. Minczuk. Mitochondrial genome engineering: the revolution may not be CRISPR-Ized. *Trends Genet.* 34:101, 2018. 29179920 10.1016/j.tig.2017.11.001 PMC5783712

[21] Gammage, P. A., J. Rorbach, A. I. Vincent, E. J. Rebar, and M. Minczuk. Mitochondrially targeted ZFNs for selective degradation of pathogenic mitochondrial genomes bearing large-scale deletions or point mutations. *EMBO Mol. Med.* 6:458, 2014. 24567072 10.1002/emmm.201303672 PMC3992073

[22] Gammage, P. A., C. Viscomi, M. L. Simard, A. S. H. Costa, E. Gaude, C. A. Powell, L. Van Haute, B. J. McCann, P. Rebelo-Guiomar, R. Cerutti, L. Zhang, E. J. Rebar, M. Zeviani, C. Frezza, J. B. Stewart, and M. Minczuk. Genome editing in mitochondria corrects a pathogenic mtDNA mutation in vivo. *Nat. Med.* 24:1691–1695, 2018. 30250142 10.1038/s41591-018-0165-9 PMC6225988

[23] Giles, R. E., H. Blanc, H. M. Cann, and D. C. Wallace. Maternal inheritance of human mitochondrial DNA. *Proc. Natl. Acad. Sci. USA*. 77:6715–6719, 1980. 6256757 10.1073/pnas.77.11.6715 PMC350359

[24] Gorman, G. S., P. F. Chinnery, S. DiMauro, M. Hirano, Y. Koga, R. McFarland, A. Suomalainen, D. R. Thorburn, M. Zeviani, and D. M. Turnbull. Mitochondrial diseases. *Nat. Rev. Dis. Prim.* 2(1):1–22, 2016. 10.1038/nrdp.2016.80 27775730

[25] Gray, M. W. Mitochondrial evolution. *Cold Spring Harb. Perspect. Biol.* 4:a011403, 2012. 22952398 10.1101/cshperspect.a011403 PMC3428767

[26] Guo, J., X. Chen, Z. Liu, H. Sun, Y. Zhou, Y. Dai, Y. Ma, L. He, X. Qian, J. Wang, J. Zhang, Y. Zhu, J. Zhang, B. Shen, and F. Zhou. DdCBE mediates efficient and inheritable modifications in mouse mitochondrial genome. *Mol. Ther. - Nucleic Acids*. 27:73–80, 2022. 34938607 10.1016/j.omtn.2021.11.016 PMC8646052

[27] Hamilton, G. The mitochondria mystery. *Nature*. 525:444–446, 2015. 26399812 10.1038/525444a

[28] Hashimoto, M., S. R. Bacman, S. Peralta, M. J. Falk, A. Chomyn, D. C. Chan, S. L. Williams, and C. T. Moraes. MitoTALEN: a general approach to reduce mutant mtDNA loads and restore oxidative phosphorylation function in mitochondrial diseases. *Mol. Ther.* 23:1592–1599, 2015. 26159306 10.1038/mt.2015.126 PMC4817924

[29] Herrmann, J. M., and W. Neupert. Protein transport into mitochondria. *Curr. Opin. Microbiol.* 3:210–214, 2000. 10744987 10.1016/S1369-5274(00)00077-1

[30] Holt, I. J., A. E. Harding, and J. A. Morgan-Hughes. Deletions of muscle mitochondrial DNA in patients with mitochondrial myopathies. *Nature*. 331(6158):717–719, 1988. 2830540 10.1038/331717a0

[31] Hoy, M. A. Molecular systematics and the evolution of arthropods. *Insect Mol. Genet.* 2013. 10.1016/B978-0-12-415874-0.00012-3. 10.1016/B978-0-12-415874-0.00012-3

[32] Jackson, C. B., D. M. Turnbull, M. Minczuk, and P. A. Gammage. Therapeutic manipulation of mtDNA Heteroplasmy: A Shifting Perspective. *Trends Mol. Med.* 26:698–709, 2020. 32589937 10.1016/j.molmed.2020.02.006

[33] Katajisto, P., J. Döhla, C. L. Chaffer, N. Pentinmikko, N. Marjanovic, S. Iqbal, R. Zoncu, W. Chen, R. A. Weinberg, and D. M. Sabatini. Asymmetric apportioning of aged mitochondria between daughter cells is required for stemness. *Science*. 348:340–343, 2015. 25837514 10.1126/science.1260384 PMC4405120

[34] Kim, C. A., and J. M. Berg. A 2.2 Å resolution crystal structure of a designed zinc finger protein bound to DNA. *Nat. Struct. Biol.* 3(11):940–945, 1996. 8901872 10.1038/nsb1196-940

[35] Lagouge, M., and N. G. Larsson. The role of mitochondrial DNA mutations and free radicals in disease and ageing. *J. Intern. Med.* 273:529–543, 2013. 23432181 10.1111/joim.12055 PMC3675642

[36] Lee, H., S. Lee, G. Baek, A. Kim, B. C. Kang, H. Seo, and J. S. Kim. Mitochondrial DNA editing in mice with DddA-TALE fusion deaminases. *Nat. Commun.* 12(1):1–6, 2021. 33608520 10.1038/s41467-021-21464-1 PMC7895935

[37] Lightowlers, R. N., P. F. Chinnery, D. M. Turnbull, N. Howell, and D. M. Turnbuu. Mammalian mitochondrial genetics: heredity, heteroplasmy and disease. *Trends Genet.* 13:450–455, 1997. 9385842 10.1016/S0168-9525(97)01266-3

[38] Liu, X., C. N. Kim, J. Yang, R. Jemmerson, and X. Wang. Induction of apoptotic program in cell-free extracts: requirement for dATP and cytochrome c. *Cell*. 86:147–157, 1996. 8689682 10.1016/S0092-8674(00)80085-9

[39] Mani, M., K. Kandavelou, F. J. Dy, S. Durai, and S. Chandrasegaran. Design, engineering, and characterization of zinc finger nucleases. *Biochem. Biophys. Res. Commun.* 335:447–457, 2005. 16084494 10.1016/j.bbrc.2005.07.089

[40] McCully, J. D., D. B. Cowan, S. M. Emani, and P. J. del Nido. Mitochondrial transplantation: from animal models to clinical use in humans. *Mitochondrion*. 2017. 10.1016/j.mito.2017.03.004. 28342934 10.1016/j.mito.2017.03.004

[41] Minczuk, M., M. A. Papworth, P. Kolasinska, M. P. Murphy, and A. Klug. Sequence-specific modification of mitochondrial DNA using a chimeric zinc finger methylase. *Proc. Natl. Acad. Sci. USA*. 103:19689, 2006. 17170133 10.1073/pnas.0609502103 PMC1750892

[42] Minczuk, M., M. A. Papworth, J. C. Miller, M. P. Murphy, and A. Klug. Development of a single-chain, quasi-dimeric zinc-finger nuclease for the selective degradation of mutated human mitochondrial DNA. *Nucleic Acids Res.* 36:3926–3938, 2008. 18511461 10.1093/nar/gkn313 PMC2475635

[43] Mingozzi, F., and K. A. High. Overcoming the Host Immune Response to Adeno-Associated Virus Gene Delivery Vectors: The Race Between Clearance, Tolerance, Neutralization, and Escape. *Annu. Rev. Virol.* 4:511–534, 2017. 28961410 10.1146/annurev-virology-101416-041936

[44] Mok, B. Y., M. H. de Moraes, J. Zeng, D. E. Bosch, A. V. Kotrys, A. Raguram, F. S. Hsu, M. C. Radey, S. B. Peterson, V. K. Mootha, J. D. Mougous, and D. R. Liu. A bacterial cytidine deaminase toxin enables CRISPR-free mitochondrial base editing. *Nature*. 583(7817):631–637, 2020. 32641830 10.1038/s41586-020-2477-4 PMC7381381

[45] Moretton, A., F. Morel, B. Macao, P. Lachaume, L. Ishak, M. Lefebvre, I. Garreau-Balandier, P. Vernet, M. Falkenberg, and G. Farge. Selective mitochondrial DNA degradation following double-strand breaks. *PLoS ONE*. 12:e0176795, 2017. 28453550 10.1371/journal.pone.0176795 PMC5409072

[46] Moscou, M. J., and A. J. Bogdanove. A simple cipher governs DNA recognition by TAL effectors. *Science*. 326:1501, 2009. 19933106 10.1126/science.1178817

[47] Nissanka, N., and C. T. Moraes. Mitochondrial DNA heteroplasmy in disease and targeted nuclease-based therapeutic approaches. *EMBO Rep.* 21:e49612, 2020. 32073748 10.15252/embr.201949612 PMC7054667

[48] Park, C. B., and N. G. Larsson. Mitochondrial DNA mutations in disease and aging. *J. Cell Biol.* 193:809–818, 2011. 21606204 10.1083/jcb.201010024 PMC3105550

[49] Peeva, V., D. Blei, G. Trombly, S. Corsi, M. J. Szukszto, P. Rebelo-Guiomar, P. A. Gammage, A. P. Kudin, C. Becker, J. Altmüller, M. Minczuk, G. Zsurka, and W. S. Kunz. Linear mitochondrial DNA is rapidly degraded by components of the replication machinery. *Nat. Commun.* 9(1):1–11, 2018. 29712893 10.1038/s41467-018-04131-w PMC5928156

[50] Pereira, C. V., S. R. Bacman, T. Arguello, U. Zekonyte, S. L. Williams, D. R. Edgell, and C. T. Moraes. mitoTev-TALE: a monomeric DNA editing enzyme to reduce mutant mitochondrial DNA levels. *EMBO Mol. Med.* 10:e8084, 2018. 30012581 10.15252/emmm.201708084 PMC6127889

[51] Persikov, A. V., J. L. Wetzel, E. F. Rowland, B. L. Oakes, D. J. Xu, M. Singh, and M. B. Noyes. A systematic survey of the Cys2His2 zinc finger DNA-binding landscape. *Nucleic Acids Res.* 43:1965–1984, 2015. 25593323 10.1093/nar/gku1395 PMC4330361

[52] Pingoud, A., G. G. Wilson, and W. Wende. Type II restriction endonucleases—a historical perspective and more. *Nucleic Acids Res.* 42:7489–7527, 2014. 24878924 10.1093/nar/gku447 PMC4081073

[53] Poulton, J., M. R. Chiaratti, F. V. Meirelles, S. Kennedy, D. Wells, and I. J. Holt. Transmission of mitochondrial DNA diseases and ways to prevent them. *PLoS Genet.* 6:e1001066, 2010. 20711358 10.1371/journal.pgen.1001066 PMC2920841

[54] Rahman, S., J. Poulton, D. Marchington, and A. Suomalainen. Decrease of 3243 A–>G mtDNA mutation from blood in MELAS syndrome: a longitudinal study. *Am. J. Hum. Genet.* 68:238–240, 2001. 11085913 10.1086/316930 PMC1234919

[55] Reddy, P., A. Ocampo, K. Suzuki, J. Luo, S. R. Bacman, S. L. Williams, A. Sugawara, D. Okamura, Y. Tsunekawa, J. Wu, D. Lam, X. Xiong, N. Montserrat, C. R. Esteban, G. H. Liu, I. Sancho-Martinez, D. Manau, S. Civico, F. Cardellach, M. Del Mar O’Callaghan, J. Campistol, H. Zhao, J. M. Campistol, C. T. Moraes, and J. C. Izpisua Belmonte. Selective elimination of mitochondrial mutations in the germline by genome editing. *Cell*. 161:459–469, 2015. 25910206 10.1016/j.cell.2015.03.051 PMC4505837

[56] Rossignol, R., B. Faustin, C. Rocher, M. Malgat, J. P. Mazat, and T. Letellier. Mitochondrial threshold effects. *Biochem. J.* 370:751–762, 2003. 12467494 10.1042/bj20021594 PMC1223225

[57] Sallevelt, S. C. E. H., J. C. F. M. Dreesen, M. Drüsedau, S. Spierts, E. Coonen, F. H. J. van Tienen, R. J. T. van Golde, I. F. M. de Coo, J. P. M. Geraedts, C. E. M. de Die-Smulders, and H. J. M. Smeets. Preimplantation genetic diagnosis in mitochondrial DNA disorders: challenge and success. *J. Med. Genet.* 50:125–132, 2013. 23339111 10.1136/jmedgenet-2012-101172

[58] Santorelli, F. M., K. Tanji, S. Shanske, and S. DiMauro. Heterogeneous clinical presentation of the mtDNA NARP/T8993G mutation. *Neurology*. 49:270–273, 1997. 9222207 10.1212/WNL.49.1.270

[59] Shokolenko, I. N., and M. F. Alexeyev. Mitochondrial DNA: a disposable genome? *Biochim. Biophys. Acta*. 1852:1805, 2015. 26071375 10.1016/j.bbadis.2015.05.016 PMC4523420

[60] Shoubridge, E. A., and T. Wai. Mitochondrial DNA and the mammalian oocyte. *Curr. Top. Dev. Biol.* 77:87–111, 2007. 17222701 10.1016/S0070-2153(06)77004-1

[61] Skladal, D., J. Halliday, and D. R. Thorburn. Minimum birth prevalence of mitochondrial respiratory chain disorders in children. *Brain*. 126:1905–1912, 2003. 12805096 10.1093/brain/awg170

[62] Smith, J., M. Bibikova, F. G. Whitby, A. R. Reddy, S. Chandrasegaran, and D. Carroll. Requirements for double-strand cleavage by chimeric restriction enzymes with zinc finger DNA-recognition domains. *Nucleic Acids Res.* 28:3361, 2000. 10954606 10.1093/nar/28.17.3361 PMC110700

[63] Spinelli, J. B., and M. C. Haigis. The multifaceted contributions of mitochondria to cellular metabolism. *Nat. Cell Biol.* 20(7):745–754, 2018. 29950572 10.1038/s41556-018-0124-1 PMC6541229

[64] Suomalainen, A., and B. J. Battersby. Mitochondrial diseases: the contribution of organelle stress responses to pathology. *Nat. Rev. Mol. Cell Biol.* 19(2):77–92, 2017. 28792006 10.1038/nrm.2017.66

[65] Szczesny, R. J., M. S. Hejnowicz, K. Steczkiewicz, A. Muszewska, L. S. Borowski, K. Ginalski, and A. Dziembowski. Identification of a novel human mitochondrial endo-/exonuclease Ddk1/c20orf72 necessary for maintenance of proper 7S DNA levels. *Nucleic Acids Res.* 41:3144, 2013. 23358826 10.1093/nar/gkt029 PMC3597694

[66] Taylor, R. W., and D. M. Turnbull. Mitochondrial DNA mutations in human disease. *Nat. Rev. Genet.* 6:389–402, 2005. 15861210 10.1038/nrg1606 PMC1762815

[67] Tools | TAL Effector Nucleotide Targeter 2.0at. https://tale-nt.cac.cornell.edu/

[68] Tuppen, H. A. L., E. L. Blakely, D. M. Turnbull, and R. W. Taylor. Mitochondrial DNA mutations and human disease. *Biochim. Biophys. Acta – Bioenerg.* 1797:113–128, 2010. 10.1016/j.bbabio.2009.09.005 19761752

[69] Wallace, D. C. A mitochondrial paradigm of metabolic and degenerative diseases, aging, and cancer: a dawn for evolutionary medicine. *Annu. Rev. Genet*. 39:359, 2005. 16285865 10.1146/annurev.genet.39.110304.095751 PMC2821041

[70] Wallace, D. C., G. Singh, M. T. Lott, J. A. Hodge, T. G. Schurr, A. M. S. Lezza, L. J. Elsas, and E. K. Nikoskelainen. Mitochondrial DNA mutation associated with Leber’s hereditary optic neuropathy. *Science*. 242:1427–1430, 1988. 3201231 10.1126/science.3201231

[71] Wang, D., P. W. L. Tai, and G. Gao. Adeno-associated virus vector as a platform for gene therapy delivery. *Nat. Rev. Drug Discov.* 18:358–378, 2019. 30710128 10.1038/s41573-019-0012-9 PMC6927556

[72] Wanrooij, S., and M. Falkenberg. The human mitochondrial replication fork in health and disease. *Biochim. Biophys. Acta*. 1797:1378–1388, 2010. 20417176 10.1016/j.bbabio.2010.04.015

[73] Wei, Y., Z. Li, K. Xu, H. Feng, L. Xie, D. Li, Z. Zuo, M. Zhang, C. Xu, H. Yang, and E. Zuo. Mitochondrial base editor DdCBE causes substantial DNA off-target editing in nuclear genome of embryos. *Cell Discov.* 8(1):1–4, 2022. 35304438 10.1038/s41421-022-00391-5 PMC8933521

[74] Yakes, F. M., and B. Van Houten. Mitochondrial DNA damage is more extensive and persists longer than nuclear DNA damage in human cells following oxidative stress. *Proc. Natl. Acad. Sci. USA*. 94:514–519, 1997. 9012815 10.1073/pnas.94.2.514 PMC19544

[75] Yamada, Y., M. Ito, M. Arai, M. Hibino, T. Tsujioka, and H. Harashima. Challenges in promoting mitochondrial transplantation therapy. *Int. J. Mol. Sci.* 2020. 10.3390/ijms21176365. 32887310 10.3390/ijms21176365 PMC7504154

[76] Yang, X., J. Jiang, Z. Li, J. Liang, and Y. Xiang. Strategies for mitochondrial gene editing. *Comput. Struct. Biotechnol. J.* 2021. 10.1016/j.csbj.2021.06.003. 34188780 10.1016/j.csbj.2021.06.003 PMC8202187

[77] Yang, Y., H. Wu, X. Kang, Y. Liang, T. Lan, T. Li, T. Tan, J. Peng, Q. Zhang, G. An, Y. Liu, Q. Yu, Z. Ma, Y. Lian, B. S. Soh, Q. Chen, P. Liu, Y. Chen, X. Sun, R. Li, X. Zhen, P. Liu, Y. Yu, X. Li, and Y. Fan. Targeted elimination of mutant mitochondrial DNA in MELAS-iPSCs by mitoTALENs. *Protein Cell*. 9:283–297, 2018. 29318513 10.1007/s13238-017-0499-y PMC5829275

[78] Zekonyte, U., S. R. Bacman, J. Smith, W. Shoop, C. V. Pereira, G. Tomberlin, J. Stewart, D. Jantz, and C. T. Moraes. Mitochondrial targeted meganuclease as a platform to eliminate mutant mtDNA in vivo. *Nat. Commun.* 12:1–11, 2021. 34050192 10.1038/s41467-021-23561-7 PMC8163834

[79] Zhang, H., S. P. Burr, and P. F. Chinnery. The mitochondrial DNA genetic bottleneck: inheritance and beyond. *Essays Biochem.* 62:225–234, 2018. 29880721 10.1042/EBC20170096

